# Impact of dexamethasone in severe COVID-19-induced acute kidney injury: a multicenter cohort study

**DOI:** 10.1186/s13613-024-01258-6

**Published:** 2024-02-13

**Authors:** Sébastien Rubin, Arthur Orieux, Mathilde Prezelin-Reydit, Antoine Garric, Yoann Picard, Nouchan Mellati, Lisa Le Gall, Antoine Dewitte, Renaud Prevel, Didier Gruson, Guillaume Louis, Alexandre Boyer, for the Groupe Recherche Rein Réanimation (G3R)

**Affiliations:** 1 Service de Néphrologie, Transplantation, Dialyse, Aphérèses, CHU de Bordeaux, Place Amélie Raba Léon, 33000 Bordeaux, France; 2 Univ. Bordeaux, INSERM, BMC, U1034, F-33600, Pessac, France; 3 Service de Médecine Intensive Réanimation, Hôpital Pellegrin, CHU de Bordeaux, Place Amélie Raba Léon, 33000 Bordeaux, France; 4 Maison du REIN-AURAD Aquitaine, Gradignan, France; 5 Univ. Bordeaux, INSERM, BPH, U1219, F-33000, Univ. Bordeaux, Bordeaux, France; 6 Service de Réanimation Polyvalente, CHR Metz, Thionville, Hôpital de Mercy, Ars-Laquenexy, France; 7 Service d’Anesthésie-Réanimation Sud, Centre Médico-Chirurgical Magellan, CHU de Bordeaux, Pessac, France; 8 Univ. Bordeaux, INSERM, CRCTB, U 1045, F-33000, Bordeaux, France

**Keywords:** Acute kidney injury, Intensive care unit, COVID-19, Dexamethasone

## Abstract

**Background:**

Acute kidney injury (AKI) in intensive care unit (ICU) patients with severe COVID-19 is common (> 50%). A specific inflammatory process has been suggested in the pathogenesis of AKI, which could be improved by dexamethasone (DXM). In a small monocenter study (*n* = 100 patients), we reported a potential protective effect of DXM on the risk of AKI. This study aimed to investigate the preventive impact of DXM on AKI in a multicenter study of patients with severe COVID-19.

**Methods:**

We conducted a multicenter study in three French ICUs from March 2020 to August 2021. All patients admitted to ICU for severe COVID-19 were included. Individuals with preexistent AKI or DXM administration before admission to ICU were excluded. While never used during the first wave, DXM was used subsequently at ICU entry, providing two treatment groups. Multivariate Cause-specific Cox models taking into account changes in ICU practices over time, were utilized to determine the association between DXM and occurrence of AKI.

**Results:**

Seven hundred and ninety-eight patients were included. Mean age was 62.6 ± 12.1 years, 402/798 (50%) patients had hypertension, and 46/798 (6%) had previous chronic kidney disease. Median SOFA was 4 [3–6] and 420/798 (53%) required invasive mechanical ventilation. ICU mortality was 208/798 (26%). AKI was present in 598/798 (75%) patients: 266/598 (38%), 163/598 (27%), and 210/598 (35%) had, respectively, AKI KDIGO 1, 2, 3, and 61/598 (10%) patients required renal replacement therapy. Patients receiving DXM had a significantly decreased hazard of AKI occurrence compared to patients without DXM (HR 0.67; 95CI 0.55–0.81). These results were consistent in analyses that (1) excluded patients with DXM administration to AKI onset delay of less than 12 h, (2) incorporating the different ‘waves’ of the COVID-19 pandemic.

**Conclusions:**

DXM was associated with a decrease in the risk of AKI in severe COVID-19 patients admitted to ICU. This supports the hypothesis that the inflammatory injury of AKI may be preventable.

**Supplementary Information:**

The online version contains supplementary material available at 10.1186/s13613-024-01258-6.

## Background

Since December 2019, coronavirus disease 2019 (COVID-19) has infected more than 570 million people, causing an excess of mortality of almost 15 million deaths worldwide that far in 2022 [1]. The severe respiratory damages requiring prolonged intubation have resulted in intensive care unit (ICU) saturation [2]. Acute kidney injury (AKI) is one of the most frequent organ damage besides pulmonary impairment. It has been observed in 25–85% of patients according to different ICU admission criteria, and an extrarenal replacement therapy (RRT) is needed in 10–15% of patients [3–6]. This leads to pressure on RRT device availability in case of ICU saturation but also an increase in the long-term burden of AKI. The need to decrease AKI incidence in severe COVID-19 is therefore critical.

Since the end of 2020, the World Health Organisation (WHO) has recommended dexamethasone (DXM) as the core treatment of respiratory damage, as its pathophysiology involves a cytokine storm. Notably, the RECOVERY trial (2020) demonstrated that DXM use in hospitalized COVID-19 patients led to a decrease in RRT need, highlighting its potential benefits in managing such cases [7]. However, whether DXM specifically impacts the incidence of COVID-19-related AKI, beyond its effects on respiratory symptoms, remains less clear. This question is particularly pertinent given the inflammatory processes suggested in COVID-19-associated-AKI pathogenesis [8]. While Lumlertgul et al. (2021) found that DXM decreased the AKI rate, Hsu et al. (2022) exhibited more kidney failure in the steroid group [3, 9]. Nevertheless, these studies were conducted during the first wave when DXM was not a standard of care and was only used in the most severe cases. After the first wave in France, DXM was used systematically, regardless of the severity of patients admitted to the ICU. In 2021, we conducted a preliminary study within the ICU of the University Hospital of Bordeaux [10]. We showed that DXM was independently associated with a decrease in the risk of AKI incidence. Still, this study suffered from its single centre design and lack of power preventing an adequate adjustment, which is critical to avoid confusion bias inherent to this kind of design. Therefore, we conducted a multicenter and most robust study to examine whether early administration of DXM could be associated with a decrease of AKI occurrence in severe COVID-19 patients.

## Methods

### Study design

This cohort study was carried out in ICUs at the University Hospital of Bordeaux (25 beds) and the Hospital of Metz-Thionville (2 units), France, from March 2020 to August 2021. Patients’ data were routinely collected in dedicated electronic health records during their hospital stay. According to French law and the French Data Protection Authority, the data handling for research purposes was declared to the Data Protection Officer of the University Hospital of Bordeaux. The French Society of Intensive Care (SRLF) ethics committee approved the study and was assigned CE SRLF 22-013. Patients (or their relatives, if any) were notified about the anonymized use of their healthcare data via a dedicated department’s booklet.

### Participants

Patients were enrolled if they were aged 18 years or older, had positive real-time reverse transcriptase–polymerase chain reaction (PCR) assays for COVID-19 or typical computed tomography findings in patients with a high clinical pretest probability of COVID-19 (for the first wave only), and had been admitted to one of the three ICUs. To assess a potential preventive effect of DXM on AKI, ICU patients with pre-existing AKI-defined Kidney Disease: Improving Global Outcomes (KDIGO) criteria at ICU admission and receiving DXM before ICU admission were excluded. All 100 patients included in the preliminary study were also part of the patient population in this current study [10].

COVID-19 waves in France were defined according to the French National Institute of Statistics and Economic Studies (INSEE): the first wave started on 2nd March 2020 and lasted until 5th July 2020, the second wave from 6th July 2020 to 4th January 2021, the third wave from 5 January 2021 to 5th July 2021 and fourth wave from 6th July 2021 to 6th September 2021 [11].

### Patient management

Patients were treated according to the standard of care. Starting from the second wave on July 2020, DXM was recommended for all patients admitted to ICU and administrated for ten days at 6 mg per day for the majority of them, whereas it was never used before. Patients were admitted based on the French Society of Intensive Care (SRLF) guideline for patients' admission in a pandemic context [12]. An initial blood test and urine test was systematically performed at admission ± 1 h.

### Outcomes

The main outcome was the risk of AKI associated with DXM. The day of AKI was the first day the patient was eligible for AKI using the KDIGO classification. The definition of KDIGO has been used to discriminate AKI Stages 1, 2, and 3: serum creatinine (SCr) and urine output were taken into account [13]. Stage 1 was defined by an SCr 1.5–1.9 times baseline or diuresis < 0.5 mL/kg/h for 6–12 h; Stage 2: SCr 2.0–2.9 times baseline or diuresis < 0.5 mL/kg/h for ≥ 12 h; Stage 3: SCr 3 times baseline, initiation of RRT, anuria ≥ 12 h or diuresis < 0.3 mL/kg/h for ≥ 24 h. AKI stage was classified using the worst SCr or diuresis during the ICU stay. Baseline SCr values corresponded to SCr values at admission in the case of normal renal function, SCr values from within six months in the case of abnormal SCr at admission or were estimated using the Modification of Diet in Renal Disease (MDRD) study equation assuming that baseline estimated glomerular filtration rate (GFR) is 75 mL/min/1.73m^2^ [14]. Patients were closely monitored for AKI development throughout their stay in the ICU, with the follow-up period concluding upon their discharge from the ICU.

Since 2017, criteria for RRT initiation have been standardized in the ICUs participating in the study, according to the delayed strategy of the Artificial Kidney Initiation in Kidney Injury (AKIKI) trial [15]. Patients were eligible for RRT whenever AKI Stage 3 occurred with clinical indications (anuria > 72 h, serum potassium > 6 mmol/L or > 5.5 mmol/L after medical correction, acute pulmonary edema, pH < 7.15 in the absence of other causes, or blood urea > 40 mmol/L). Recovery from AKI was assessed and defined as a return of SCr to ≤ 125% above baseline SCr for alive and non-dependent RRT patients [16].

Other outcomes include the ICU length of stay, which was only calculated for patients discharged from the ICU: and the intubation length which was only calculated for the subgroup of successfully extubated patients. If a patient ever needed to be re-intubated after having been weaned from the ventilator, the intubation length was calculated as the sum of the different periods during which invasive mechanical ventilation was required.

### Exposure variables

Information on the date of the first COVID-19 symptoms and medical history of hypertension, diabetes, chronic ischaemic heart disease, stroke, chronic obstructive pulmonary disease, asthma, and obstructive sleep apnea were collected using prospectively recorded data and patient/relative questioning. The SARS-CoV-2 variant was also collected when available. Unavailable data of variants were imputed to the current predominant epidemic variant at the time of admission. Pre-admission exposure to renin–angiotensin system inhibitors was considered if the patient had taken the drug the week before admission. Weight and height were measured at ICU admission. All other healthcare data were collected using Metavision ICU (iMDsoft) and IntelliSpace Critical Care and Anesthesia software (ICCA, Philips). Patients chronically exposed to immunosuppressive drugs or suffering from hematological malignancies were considered immunosuppressed. The worst PaO2/FiO2 ratio was calculated in the first 24 h post-ICU admission. Chronic kidney disease (CKD) was defined as an estimated GFR of < 60 mL/min/1.73 m^2^, using the Chronic Kidney Disease–Epidemiology Collaboration (CKD–Epi) corresponding to CKD Stage 3 or more according to the KDIGO classification [17]. Minimum diuresis was defined by the minimum diuresis/kg/h used for the KDIGO classification. All blood or urine biological assessments were usually standardized in ICUs participating in the study and systematically collected. The day of DXM administration was noted on the first day of injection.

### Statistical analysis

Descriptive statistics incorporated mean ± standard deviation (SD) or median [interquartile range (IQR)] for variables that did not fit a normal distribution. Quantitative variables were compared using a *t* test, and qualitative variables were compared using Fisher’s exact test when only two variables were studied; Pearson’s Chi-squared test was used for more variables.

The temporal aspect of COVID-19 waves was a significant factor that influenced the strategy of DXM administration. During the initial wave, DXM administration was nil (probability = 0), while during subsequent waves, DXM was administered to the majority of patients. This temporal change precluded the use of propensity score matching or adjustment.

Cumulative incidences were estimated in each treatment group using the Aalen–Johansen estimator to account for competing risks [18, 19]. The two groups were compared using the Gray test. To estimate the adjusted hazard ratio for AKI occurrence between patients who received DXM and those who did not, while accounting for the competing risk of death, we employed a cause-specific Cox model. This model was adjusted for age (in years), sex (male vs female), BMI (in kg/m^2^), CKD (yes vs no), HTN (yes vs no), diabetes (yes vs no), immunodepression (yes vs no), SAPS II (in units), invasive mechanical ventilation during the first 24 h (yes vs no), intravenous fluid therapy during the first 24 h (per L), catecholamine use (yes vs no), and COVID-19 variant (variant XX).

The primary exposure variable was the use of DXM. Adjustment variables were chosen based on their association with AKI from previous studies and from our univariate analysis. These variables were determined to be mediators in the relationship between DXM and the outcome rather than confounders, as they do not influence the decision to administer DXM. The starting point (T0) was the ICU admission. The time axis was the delay from ICU admission. The outcome was the occurrence of AKI, with censoring events being ICU discharge or death. The proportional hazards assumption was verified using Schoenfeld residuals. Variables for adjustment were predefined. We initially chose not to include the ‘center’ as an adjustment variable in the primary analysis, deeming it more pertinent to account for a center effect when the intervention’s quality (here, the administration of DXM) might be influenced by the center itself. Nonetheless, due to the distinctive challenges of COVID-19 and variations in the pandemic’s progression across centers, we included the center as a factor in a sensitivity analysis (Model 2). A separate sensitivity analysis excluded patients with a delay of less than 12 h between the administration of DXM and the onset of AKI, and this did not alter the principal findings (Model 3). In addition, in our sensitivity analysis, we integrated the ‘waves’ of COVID-19 as a confounding variable in our cause-specific Cox model (Model 4). We also examined if these waves modified the effect of DXM on AKI occurrence by introducing an interaction term between the waves and DXM administration. This was done to assess whether the observed association between DXM and decreased AKI incidence could be influenced by the evolving ICU practices over the different COVID-19 waves (Model 3). We did not adjust on waves in the first models because we preferred adjust on variants (adjusting on both would have led to over adjustment because of colinearity between both criteria).

A *p* value of less than 0.05 was considered statistically significant (double-sided).

## Results

### Patient characteristics

**Fig. 1:**
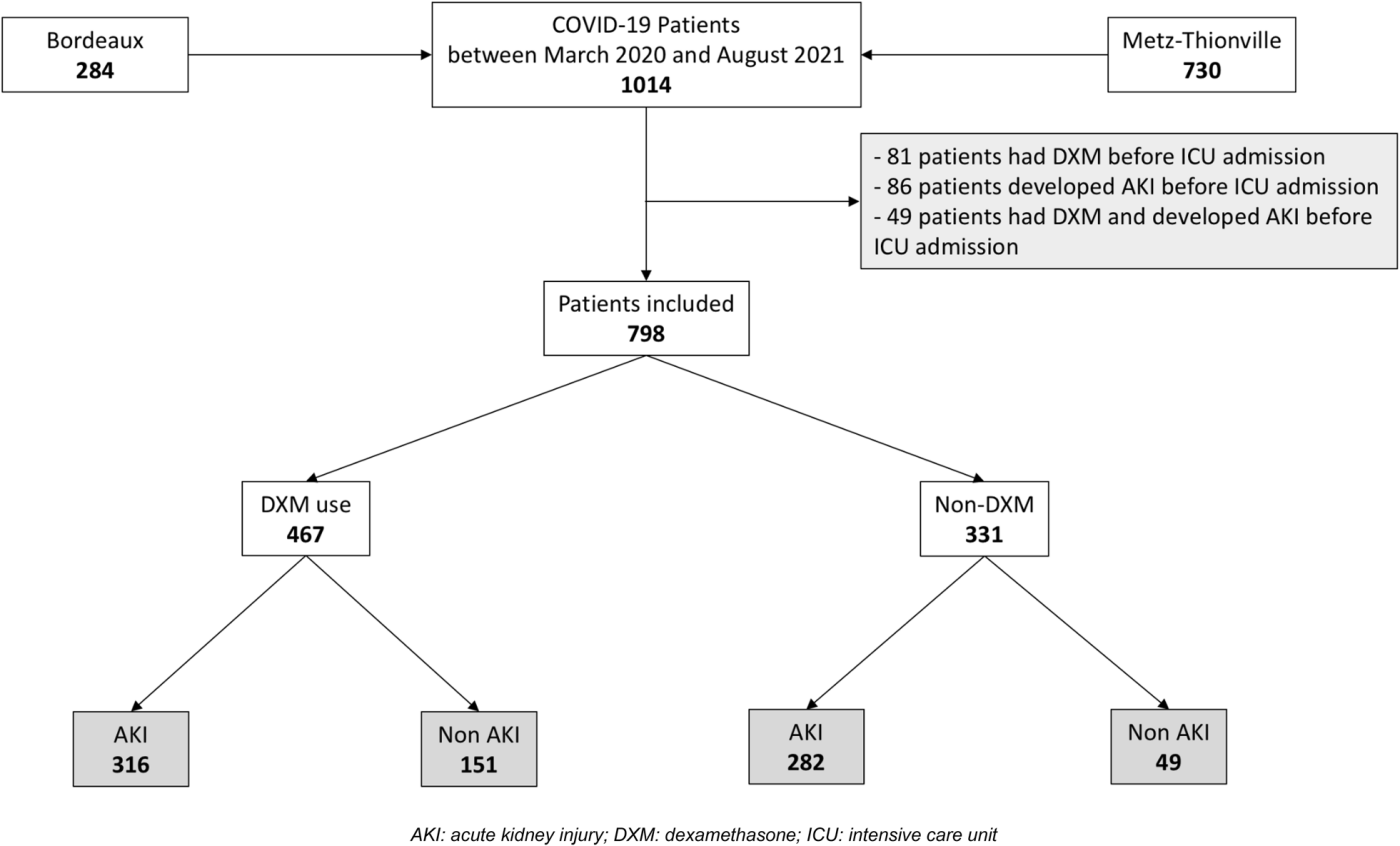
Flow chart. *AKI* acute kidney injury, *DXM* dexamethasone, *ICU* intensive care unit

From March 2020 to August 2021, 1014 COVID-19 patients met inclusion criteria. Two-hundred and eighty-four (284/1014, 28%) patients were admitted to the University Hospital of Bordeaux, and 730/1014 (72%) to the Regional Hospital of Metz-Thionville for severe COVID-19. Among them, 107/1014 (11%) patients received DXM before ICU admission, and 122/1014 (12%) developed AKI before ICU admission and were excluded from the study, resulting in a total of 798 patients included (Fig. 1).

The mean age was 62.6 ± 12 years, with most males [560/798 (70%)]. The mean body mass index (BMI) was 30.6 ± 6.6 kg/m^2^, 402/798 (50%) patients had hypertension, 46/798 (6%) had previous CKD, and 231/798 (29%) had diabetes. Details on all baseline characteristics are presented in Table 1.

**Table 1:** Patient characteristics

Characteristics of patients	All patients (*n* = 798)	No DXM (*n* = 331)	DXM (*n* = 467)	*p* value
Baseline characteristics				
Bordeaux, *n* (%)	162 (20%)	53 (16%)	109 (23%)	0.012
Metz-Thionville,* n* (%)	636 (80%)	278 (84%)	358 (77%)	0.011
SARS-CoV-2 variant				
Wuhan, *n* (%)	516 (65%)	295 (89%)	221 (47%)	< 0.001
Alpha, *n* (%)	154 (19%)	21 (6%)	133 (28%)	
Beta, *n* (%)	104 (13%)	15 (4%)	89 (19%)	
Gamma, *n* (%)	2 (0.3%)	0 (0%)	2 (0.4%)	
Delta, *n* (%)	22 (3%)	0 (0%)	22 (5%)	
Males, *n* (%)	560 (70%)	248 (78%)	318 (68%)	0.127
Age (years), mean ± SD	63 ± 12	64 ± 12	62 ± 12	0.037
BMI (kg/m^2^), mean ± SD	30.6 ± 6.6	30.7 ± 6.4	30.6 ± 6.7	0.88
Chronic kidney disease, *n* (%)	46 (6%)	22 (7%)	24 (5%)	0.368
Basal SCr (µmol/L), median [IQR] *(missing values, n* = *470)*	71 [59–88]	75 [63–92]	70 [58–84]	0.48
Hypertension, *n* (%)	402 (50%)	170 (51%)	232 (50%)	0.64
Diabetes, *n* (%)	231 (29%)	91 (27%)	140 (30%)	0.445
Immunosuppression, *n* (%)	39 (5%)	15 (4%)	24 (5%)	0.695
SAPS II, median [IQR]	35 [29–45]	40 [29–52]	33 [28–41]	< 0.01
SOFA, median [IQR]	4 [3–6]	5 [4–8]	4 [3–5]	< 0.01
Non-renal SOFA, median [IQR]	4 [3–5]	3 [4–7]	4 [3–5]	0.05
Time between ICU admission and DXM administration	NA	NA	1 [1–1]	
ICU management				
Crystalloid infusion during the first 24 h (L)	1.7 ± 1.2	2.2 ± 1.4	1.7 ± 1.2	< 0.001
Catecholamine use during the first 24 h, *n* (%)	156 (19%)	96 (29%)	60 (12%)	< 0.001
Catecholamine use during ICU hospitalization, *n* (%)	372 (47%)	210 (63%)	162 (35%)	< 0.001
Invasive mechanical ventilation, *n* (%)	420 (53%)	215 (70%)	205 (43%)	< 0.001
Worst PaO_2_/FiO_2_, median [IQR]	105 [74–146]	106 [77–155]	103 [72–138]	0.11
Prone position, *n* (%)	470 (59%)	169 (51%)	301 (64%)	< 0.001

*BMI* body mass index, *DXM* dexamethasone, *FiO*_*2*_ inspired fraction of oxygen, *ICU* intensive care unit, *IQR* interquartile range, *PaO*_*2*_ arterial partial pressure of oxygen, *SAPS II* simplified acute physiology score, *Scr* serum creatinine, *SD* standard deviation, *SOFA* sequential organ failure assessment score

Among all descriptive variables in Table 1, only basal SCr variable had missing values. SCr missing values were estimated using the Modification of Diet in Renal Disease (MDRD) study equation assuming that baseline eGFR is 75 mL/min/1.73 m^2^

Intubation was required for 420/798 (53%) patients. During the first 24 h, the worst PaO_2_/FiO_2_ was 105 [74–146] *n* = 300). Norepinephrine was needed for 372/798 (47%) patients. The median Sequential Organ Failure Assessment (SOFA) was 4 [3–6]). Details on all comparison characteristics between Bordeaux and Metz-Thionville Hospitals are presented in Additional file 1: Table S1 and patients’ characteristics according to the wave in Additional file 1: Table S2.

### DXM administration after ICU admission

Of the 798 patients, 467/798 (68%) had DXM administration. Among the 331 patients who did not receive DXM, 7/331 (2%) patients were administrated hydrocortisone hemisuccinate within the first 48 h. The median SOFA was 4 [3–5] in DXM users vs. 5 [4–8] in no-DXM group (*p* < 0.01). Invasive mechanical ventilation was needed in 205/467 (43%) patients in DXM group vs. 215/331 (70%) in non-users (*p* < 0.001). Fewer individuals died in the DXM use group with 99/467 (21%) death vs. 109/331 (33%) in non-DXM-users (*p* < 0.001). Details on all characteristics associated with DXM use are presented in Table 1.

### Outcomes

Among all included patients, within a median delay of 3.2 days (IQR 25–75 1.2–8), 598/798 (75%) subjects developed an AKI, of which 331/598 (85%) in the no-DXM group and 467/598 (67%) in the DXM group (*p* < 0.001). AKI-Stage 1, 2 and 3 occurred in 225/798 (38%), 163/798 (27%) and 210/798 (35%) patients, respectively, with difference only in stage 3 AKI between the two groups. RRT was needed in 20/467 (6%) vs. 41 (12%) patients in DXM vs. no-DXM group, respectively (*p* < 0.001). The median length of intubation was 12 [6–20] days 9 [5–19] days for ICU stay. Two hundred eight patients died during their ICU stay (208/798) (26%). Details on outcomes are presented in Table 2.

**Table 2:** Outcomes

	All patients (*n* = 798)	Non DXM (n = 331)	DXM (*n* = 467)	*p* value
Acute kidney injury*, *n* (%)	598 (75%)	282 (85%)	316 (67%)	< 0.001
Defined using SCr, *n* (%)	345 (58%)	198 (70%)	147 (47%)	< 0.001
Defined using diuresis criterion, *n* (%)	552 (92%)	249 (88%)	303 (96%)	0.030
AKI Stage 1, *n* (%)	225 (38%)	100 (35%)	125 (40%)	0.287
AKI Stage 2, *n* (%)	163 (27%)	73 (26%)	90 (28%)	0.337
AKI Stage 3, *n* (%)	210 (35%)	109 (39%)	101 (32%)	0.001
Renal replacement therapy, *n* (%)	61 (10%)	41 (15%)	20 (6%)	< 0.001
SCr at admission (µmol/L), median [IQR] (missing values, *n* = 94)	73 [58–95]	79 [63–110]	69 [56–86]	< 0.01
Length of intubation (days), median [IQR]	12 [6–20]	13 [6–22]	13 [7–21]	0.6
Length of sedation (days), median [IQR]	10 [3–18]	10 [3–19]	8 [3–17]	0.13
ICU length of stay (days), median [IQR]	9 [5–19]	11 [5–24]	8 [4–17]	< 0.01
ICU death, *n* (%)	208 (26%)	109 (33%)	99 (21%)	< 0.001

*AKI* acute kidney injury, *DXM* dexamethasone, *IQR* interquartile range, *SCr* serum creatinine

*According to KDIGO classification, only one criterion (serum creatinine rise or urine output decline) must be fulfilled

**Fig. 2:**
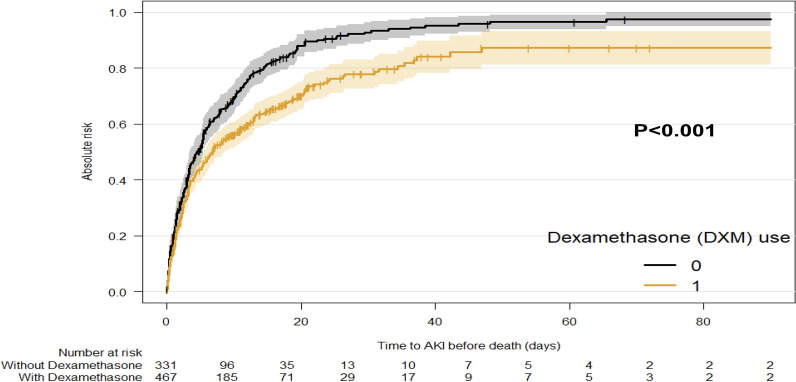
Cumulative incidence of AKI. The cumulative incidence of AKI is computed considering death as a competing event. This means that the time to AKI is censored if death occurs prior to AKI. (*p* value for Gray’ test)

Figure 2 presents the cumulative incidence of AKI from ICU admission taking into account the competing risk of death.

### Risk of AKI associated with DXM

In comparison with non-AKI patients, AKI patients were older (63 ± 11 years vs. 60 ± 13 years; *p* = 0.013), had a higher BMI (31 ± 6.9 kg/m^2^ vs. 29.7 ± 5.7 kg/m^2^; *p* = 0.022) a more frequent hypertension (314/598 (52%) cases vs. 88/200 (44%); *p* = 0.041), a higher SOFA (5 [4–7] vs. 3 [3–4]; *p* < 0.01), a more frequent norepinephrine use at admission (138/598 (23%) vs. 18/200 (9%); *p* < 0.001) and need for invasive mechanical ventilation (369/598 (61%) vs. 51/200 (25%); *p* < 0.001). Mortality in ICU was also higher, 186/598 (31%) vs. 22/200 (11%) deceased patients in AKI versus non-AKI group (*p* < 0.001). Comparaison of AKI and non-AKI patients is presented in Additional file 1: Table S3.

At any time after entry into the ICU, patients receiving DXM had a significantly decreased hazard of AKI occurrence compared to those not receiving DXM: HR 0.67; 95% CI (0.55–0.81) (Model 1, Table 3). This HR remained significant after adjustment for the center: HR 0.76; 95% CI (0.62–0.92). When excluding patients with a delay of less than 12 h between DXM administration and AKI onset, the hazard ratio was similar to that in Model 1, with an HR of 0.62; 95% CI (0.49–0.77) (Model 3).

**Table 3:** Cause-specific Cox model

Association between dexamethasone use and occurrence of AKI in patients admitted in an ICU for severe COVID-19 infection. Results of cause-specific Cox models		
	HR*	95 CI
Dexamethasone use (vs no use): Model 1	0.67	0.55–0.81
Model 2 (Model 1 adjusted for center)	0.76	0.62–0.92
Model 3 (Excluding patients with a delay of less than 12 h from the administration of DXM and the AKI)	0.62	0.49–0.77
Model 4 (Model 1 adjusted for wave rather than COVID variant)	0.75	0.57–1.00

Model 3: Results of a cause-specific Cox model excluding patients with a delay of less than 12 h from the administration of DXM and the AKI

Model 4: Results of a cause-specific Cox model adjusted for wave rather than COVID variant

*Adjusted for age (in years), sex (male/female), BMI (in kg/m^2^), CKD before ICU admission (yes vs no), hypertension before ICU admission (yes vs no), history of diabetes before ICU admission (yes vs no), immunodepression before ICU admission (yes vs no), invasive mechanical ventilation in the first 24 h (yes vs no), intravenous fluid therapy in the first 24 h (per L), Catecholamine use in the first 24 h (yes vs no), SAPS II (per unit), the COVID variant (Wuhan/Alpha/Beta/Gamma/Delta) or wave (1/2/3/4) (only for model 4) and center (model 2)

*BMI* body mass index, *CKD* chronic kidney disease, *SAPS II* simplified acute physiology score

Evolving ICU management practices during the different COVID-19 waves are presented in Additional file 1: Table S2. Incorporating the 'waves' of COVID-19 in our sensitivity analysis (Model 4), we found that the trends of reduced AKI incidence with DXM use remained favorable: HR 0.75; 95% CI (0.57, 1.00). Moreover, the effect of DXM on AKI incidence was not modified by the different ‘waves’ (p for the interaction between waves and DXM = 0.51).

In a sensitivity analysis conducted to ensure the robustness of our findings, we considered the 79 patients who did not initially receive DXM, although they were included after the first wave (Additional file 1: Table S1), as DXM recipients. This analysis, detailed in Additional file 1: Table S4, aimed to assess the potential impact of treatment classification on our results. We found a consistent association between DXM use and a decreased risk of AKI with an HR of 0.63; 95% CI (0.51–0.79). In a second sensitivity analysis, which included only patients with baseline serum creatinine data (not estimated baseline creatinine data), (*n* = 328), we found that early administration of DXM within 24 h significantly reduced the risk of acute kidney injury [adjusted HR 0.648, 95% CI (0.45–0.94)] (Additional file 1: Table S5).

## Discussion

In this multicentre ICU study, the incidence of COVID-19-associated-AKI was 75%. AKI was severe, with 35% of AKI stage 3. Our analysis revealed that the use of DXM was significantly and independently associated with a decrease in the risk of AKI development. This was shown in the primary model, as well as consistently upheld across various sensitivity analyses. As for other factors, intravenous fluid therapy within the first 24 h post ICU admission had a protective impact, reducing AKI incidence.

The pathogenesis of AKI in COVID-19 ICU patients involves several factors. Direct viral toxicity on renal cells remains controversial since renal tropism by SARS-CoV-2 is still arguable. Furthermore, ICU patients may present nonspecific factors like absolute or relative hypovolemia, nephrotoxicity of some drugs, and venous congestion due to mechanical ventilation [20, 21]. Moreover, systemic inflammation due to cytokine storm is associated with endothelial damage and complement activation, leading to acute tubular necrosis, collapsing glomerulopathy, or thrombotic microangiopathy [8]. By its anti-inflammatory effect, DXM might attenuate this cytokine storm and decrease the renal damage of COVID-19 [22]. This effect was studied in a preventive context, since the patients included in the analysis did not have AKI before DXM administration or admission to ICU.

The rate of AKI (75%) is consistent with our first assessment during the first wave, and recent other studies (AKI incidence of 50–80%) (6,9,21–23). AKI was characterised by the clear predominance of the diuresis criterion collected exhaustively, representing about 90% of cases, compared with 50% for the creatinine criterion. Indeed, Lumlertgul et al. described an AKI incidence of 76% among 313 severe COVID-19 patients [3]. In a more recent large multicenter Belgian study, Schauebroek et al. (2022) observed that 85% of COVID-19 patients admitted to ICU developed AKI, with the same RRT incidence (10%) [23]. These two studies used combined SCr and diuresis criteria.

The role of steroids on COVID-19-associated AKI has already been assessed in several studies with different outcomes. While Lumlertgul et al. (2021) found that DXM improved AKI rate, Hsu et al. (2022) exhibited more kidney failure in the steroid group [9]. Moreover, regarding RRT outcomes, while Horby et al. in Recovery Study brought out an improvement in the need for hemodialysis, Sullivan et al. (2021) exhibited a higher RRT incidence in the steroid group [5]. Nevertheless, those studies were conducted during the first wave when DXM was not a standard of care and steroids were only reserved for the most critical cases among ICU patients. The observed trend in Model 4—which included the ‘waves’ of COVID-19—suggests that the effect of DXM is consistent across the different periods of the pandemic. This finding reinforces the beneficial role of DXM use in reducing AKI incidence, even when taking into account the potential impact of evolving practices. This finding strongly implies a direct protective effect of DXM on the kidneys, though we cannot exclude that part of this benefit may be due to a reduced need for mechanical ventilation. Future studies exploring the intricate mechanisms underlying these observations would be both intriguing and illuminating.

Specific treatments for Acute Respiratory Distress Syndrome (ARDS), such as inhaled Nitric Oxide (iNO), might influence the risk of AKI. However, in our cohort, only a small proportion of patients (approximately 11%) received iNO. Regarding PEEP, while elevated intrathoracic pressures associated with higher PEEP levels might impair venous return and contribute to kidney congestion, we chose not to focus on specific PEEP values in our analysis. Instead, our study looked at the broader factor of whether patients were intubated and mechanically ventilated. We based this decision on the rationale that the overall condition of being mechanically ventilated could have a more significant impact on patients’ hemodynamic and renal risk than variations in PEEP levels. This approach was designed to provide a clearer understanding of respiratory support’s influence on renal function without the complexity and potential confusion of interpreting specific PEEP values, which could change frequently during a patient’s ICU stay. Future studies with detailed, prospective data collection on PEEP levels, tidal volumes, and pulmonary compliance might offer deeper insights into these specific aspects of mechanical ventilation and their effects on renal function in critically ill patients. Invasive ventilation in COVID-19 was also associated with AKI incidence in those assessments [3, 5, 24]. Our results do not apply to all forms of ICU–AKI. Renal damage associated with COVID-19 is characterized by severe systemic inflammation.

Moreover, the protective impact of DXM cannot apply to non-ICU patients who have no indication for DXM, according to the recommendations [7]. Furthermore, the preventive effect of DXM does not apply to other COVID-19 nephropathies, like focal segmental glomerulosclerosis or thrombotic microangiopathy, which have distinct pathogenesis [25–27]. Whether DXM could prevent AKI in other sepsis needs to be assessed in specific controlled studies [28].

In our main analysis, we opted to adjust for the different COVID-19 variants rather than the waves of the pandemic, given the documented variations in pathogenicity amongst the variants which continuously evolved throughout our study period. We acknowledge that not adjusting for the pandemic waves might be a limitation; however, it was not feasible to adjust for both since these variables are highly collinear. Nevertheless, in a sensitivity analysis (Model 4), where we adjusted for the waves, we observed a similar protective effect of DXM.

The study’s strengths include a large number of patients. This results in a significant power-providing adjustment with 13 different variables and decreasing albeit not eliminating confusion bias. By excluding patients who already had DXM or AKI before admission, we limited selection bias and focused on the preventive impact of DXM before AKI. This study also has some limitations. First, since DXM became a standard of care at the end of the first wave, most of our unexposed patients were from the first wave of COVID-19 when the ICU saturation was critical. Second, more severe patients in Metz-Thionville hospitals marked a centre effect due to the larger first wave in Eastern France. Yet, the standard of care was applied in both hospitals, so the differences are due to the subjects’ severity, as demonstrated by the higher SOFA. Third, accurate baseline SCr results were available in only 40% (328 cases). In the remaining cases, basal SCr was estimated using the Modification of Diet in Renal Disease (MDRD) study equation, assuming that baseline eGFR was 75 mL/min/1.73 m^2^. This approach may introduce classification bias. However, it is a conventionally employed method. Importantly, this method might underestimate the incidence of AKI (a threshold of 75 mL/min might lead to a 50% underestimation of AKI incidence as the mean/median GFR in relevant populations approximates 100 mL/min). This could result in the exclusion of CKD patients incorrectly considered as prior AKI [29]. Lastly, in considering our findings, it is essential to acknowledge the potential residual confounding due to differences in treatment practices and hospital conditions across COVID-19 waves. Despite our efforts to adjust for these variables, factors such as the evolution of intubation practices may not be entirely accounted for by traditional severity scores, possibly impacting our interpretation of severity markers and outcomes across different patient cohorts.

## Conclusion

In this multicenter cohort study, our findings indicate a potential association between DXM use and a decreased risk of developing severe COVID-19-associated AKI. These results lend support to the idea that the inflammatory injury of AKI in the context of COVID-19 might be partially mitigable through DXM administration. However, considering the complex and evolving nature of COVID-19 treatment practices, these conclusions should be interpreted with an awareness of potential confounding factors.

### Supplementary Information

**Additional file 1.** **Table S1:** Bordeaux and Metz-Thionville Hospitals comparison. **Table S2:** Patient's characteristics according to the wave. **Table S3:** Comparison of AKI and non AKI patients. **Table S4:** Association between dexamethasone use and occurrence of AKI in patients admitted in an ICU for severe COVID-19 infection. Sensitivity analysis: results of a cause-specific Cox model assuming that all patients admitted after 5th July 2020 received DXM. **Table S5:** Association between dexamethasone use and occurrence of AKI in patients admitted in an ICU for severe COVID-19 infection: sensitivity analysis, which included only patients with baseline serum creatinine data (not estimated baseline creatinine data), (n=328).

## Data Availability

The data set used and analyzed for the current study is available from the corresponding author upon reasonable request.

## Abbreviations

AKI: Acute kidney injury
AKIKI: Artificial kidney initiation in kidney injury
BMI: Body mass index
CKD-Epi: Chronic Kidney Disease Epidemiology Collaboration
CKD: Chronic kidney disease
COVID-19: Coronavirus disease 2019
DXM: Dexamethasone
FiO_2_: Inspired fraction of oxygen
GFR: Glomerular filtration rate
ICU: Intensive care unit
INSEE: Institute of Statistics and Economic Studies
IQR: Interquartile range
KDIGO: Kidney disease: improving global outcomes
MDRD: Modification of diet in renal disease
PaO_2_: Arterial partial pressure of oxygen
PaO_2_/FiO_2_: Partial pressure of oxygen/fraction of inspired oxygen ratio
PCR: Polymerase chain reaction
RRT: Renal replacement therapy
SAPS II: Simplified acute physiology score
SCr: Serum creatinine
SD: Standard deviation
SOFA: Sequential organ failure assessment score
SRLF: Société de Réanimation de Langue Française (French Society of Intensive Care)
WHO: World Health Organisation
