# Supplementary material for: Impact of dexamethasone in severe COVID-19-induced acute kidney injury: a multicenter cohort study
Source: Ann Intensive Care. 2024 Feb 13;14:26. doi: 10.1186/s13613-024-01258-6 (PMC10864230; doi:10.1186/s13613-024-01258-6)
Supplement: Supplementary file 1 — Additional file 1. Table S1: Bordeaux and Metz-Thionville Hospitals comparison. Table S2: Patient's characteristics according to the wave. Table S3: Comparison of AKI and non AKI patients. Table S4: Association between dexamethasone use and occurrence of AKI in patients admitted in an ICU for severe COVID-19 infection. Sensitivity analysis: results of a cause-specific Cox model assuming that all patients admitted after 5th July 2020 received DXM. Table S5: Association between dexamethasone use and occurrence of AKI in patients admitted in an ICU for severe COVID-19 infection: sensitivity analysis, which included only patients with baseline serum creatinine data (not estimated baseline creatinine data), (n=328). [file 13613_2024_1258_MOESM1_ESM.docx]

**Table S1: Bordeaux and Metz-Thionville Hospitals comparison**

| Characteristics of patients | Bordeaux  (*n* = 162) | Metz-Thionville  (*n* = 636) | *p* value |
| --- | --- | --- | --- |
| Males, *n* (%) | 120 (74%) | 440 (69%) | 0.249 |
| Age (years), mean ± SD | 61.6 ± 11.3 | 62.8 ± 12.3 | 0.263 |
| BMI (kg/m²), mean ± SD | 30.1 ± 5.7 | 30.8 ± 6.8 | 0.185 |
| CKD, *n* (%) | 11 (7%) | 35 (5%) | 0.571 |
| Hypertension, *n* (%) | 86 (53%) | 316 (50%) | 0.482 |
| Diabetes, *n* (%) | 45 (29%) | 186 (29%) | 0.771 |
| Immunosuppression, *n* (%) | 20 (12%) | 19 (3%) | **< 0.001** |
| SAPS II, median [IQR] | 36 [29-49] | 35 [29-45] | 0.38 |
| SOFA, median [IQR] | 4 [3-5] | 4 [3-7] | **< 0.01** |
| Non-renal SOFA, median [IQR] | 3.5 [3-5] | 4 [3-5] | 0.10 |
| Catecholamine use during the 1st day of admission, *n* (%) | 15 (9%) | 141 (22%) | **<0.001** |
| Invasive mechanical ventilation, *n* (%) | 85 (52%) | 335 (53%) | 0.96 |
| Length of intubation (days), median [IQR] | 15 [8-29] | 11.3 [5-20] | **< 0.01** |
| Length of sedation (days), median [IQR] | 7.5 [0-17] | 10 [3-18] | **0.03** |
| Prone position, *n* (%) | 75 (46%) | 395 (62%) | **< 0.001** |
| Catecholamine use, *n* (%) | 64 (39%) | 308 (48%) | **0.043** |
| Dexamethasone, *n* (%) | 109 (67%) | 358 (56%) | **0.012** |
| Lymphocytes (per G/L), mean ± SD | 1.1 ± 4.6 | 1.0 ± 4.0 | 0.706 |
| SARS-CoV-2 Variant | | | |
| *Wuhan, n (%)* | 103 (64%) | 416 (65%) | **< 0.001** |
| *Alpha, n (%)* | 45 (28%) | 109 (17%) |  |
| *Beta, n (%)* | 0 (0%) | 104 (16%) |  |
| *Gamma, n (%)* | 1 (0.6%) | 1 (0.2%) |  |
| *Delta, n (%)* | 13 (8%) | 9 (1.4%) |  |
| ICU length of stay (days), median [IQR] | 10 [5-19] | 9 [4-19] | 0.37 |
| ICU death, *n* (%) | 24 (15%) | 184 (29%) | **< 0.001** |
| Renal outcomes |  | | |
| Acute kidney injury, *n* (%) | 62 (38%) | 536 (84%) | **< 0.001** |
| *Defined using SCr criterion, n (%)* | 52 (37%) | 293 (46%) | 0.06 |
| *Deﬁned using diuresis criterion, n (%)* | 46 (33%) | 506 (80%) | **< 0.001** |
| SCr at admission (µmol/L), median [IQR] | 73 [58-87] | 73 [58-97] | 0.16 |
| AKI Stage 1, *n* (%) | 25 (19%) | 200 (37%) | **< 0.001** |
| AKI Stage 2, *n* (%) | 18 (14%) | 145 (27%) | **0.001** |
| AKI Stage 3, *n* (%) | 19 (14%) | 191 (26%) | **< 0.001** |
| Acute kidney disease, *n* (%) | 22 (35%) | 291 (54%) | **0.007** |
| Renal replacement therapy, *n* (%) | 9 (6%) | 52 (8%) | 0.321 |

*AKI: acute kidney injury; BMI: body mass index; FiO2: inspired fraction of oxygen; ICU: intensive care unit; PaO2: arterial partial pressure of oxygen; SAPS II: Simplified Acute Physiology Score; SOFA: Sequential Organ Failure Assessment Score*

**Table S2 : Patient's characteristics according to the wave**

| **Characteristics of patients** | **Wave 1** | **Wave 2** | **Wave 3** | **Wave 4** | ***p* value** |
| --- | --- | --- | --- | --- | --- |
|  | **(N=253)** | **(N=247)** | **(N=267)** | **(N=31)** |  |
| Dexaméthasone Use | 0 (0%) | 204 (83%) | 232 (87%) | 30 (97%) | P<0.01 |
| Bordeaux, *n* (%) | 52 (21%) | 55 (22%) | 36 (14%) | 19 (61%) | P<0.01 |
| Metz-Thionville, *n* (%) | 201 (79%) | 192 (78%) | 231 (86%) | 12 (39%) | P<0.01 |
| Age (years), mean ± SD | 63.5±12.5 | 65.9± 10.3 | 59.7±12.3 | 56 ±12.7 | P<0.01 |
| Males, *n* (%) | 184 (73%) | 181 (73%) | 175 (66%) | 20 (65%) | P=0.16 |
| BMI (kg/m²), mean ± SD | 30.7±6.4 | 30.2±6.8 | 31.2±6.3 | 29.9±8.2 | P=0.15 |
| Chronic kidney disease, *n* (%) | 16 (6%) | 18 (7.3%) | 11 (4.1%) | 1 (6%) | P=0.41 |
| Basal SCr (µmol/L), median [IQR] *(missing values, n = 470)* | 75 [62 92] | 71.0 [58-86] | 74 [58-84] | 62 [58- 80] | P=0.30 |
| Hypertension, *n* (%) | 136.0 (53.8%) | 132.0 (53.4%) | 119.0 (44.6%) | 15.0 (48.4%) | P=0.13 |
| Diabetes, *n* (%) | 70.0 (27.7%) | 87.0 (35.2%) | 66.0 (24.7%) | 8.0 (25.8%) | P=0.06 |
| Immunosuppression, *n* (%) | 11.0 (4.3%) | 13.0 (5.3%) | 11.0 (4.1%) | 4.0 (12.9%) | P=0.18 |
| SAPS II, median [IQR] | 40 [29-53] | 36 [30-43] | 32 [26-40] | 32 [27-42] | P<0.01 |
| SOFA, median [IQR] | 5 [4-8] | 3 [4-6] | 3 [4-5] | 3 [3-4] | P<0.01 |
| Crystalloid infusion during the ﬁrst 24 h (L) | 2.4±1.3 | 1.6±1.0 | 1.4±1.2 | 1.6±1.0 | P<0.01 |
| Catecholamine use during the first 24h, *n* (%) | 78.0 (30.8%) | 37.0 (15.0%) | 36.0 (13.5%) | 5.0 (16.1%) | P<0.01 |
| Catecholamine use during ICU hospitalization, *n* (%) | 170.0 (67.2%) | 96.0 (38.9%) | 99.0 (37.1%) | 7.0 (22.6%) | P<0.01 |
| Invasive mechanical ventilation, *n* (%) | 182.0 (71.9%) | 105.0 (42.5%) | 122.0 (45.7%) | 11.0 (35.5%) | P<0.01 |
| Worst PaO_2_/FiO_2_, median [IQR] | 106 [77-156] | 106 [70-145] | 102 [74-132] | 118 [86-149] | P=0.16 |
| Prone position, *n* (%) | 127.0 (50.2%) | 139.0 (56.3%) | 188.0 (70.4%) | 16.0 (51.6%) | P<0.01 |
| Length of intubation (days), median [IQR] | 12 [5-22.0] | 15 [8- 21] | 12 [6-19] | 7 [3-12] | P=0.17 |
| Length of sedation (days), median [IQR] | 10 [4-19] | 11 [5-20] | 11 [4-19] | 5 [2-13] | P=0.30 |
| Acute Kidney Injury, *n (*%) | 217 (86%) | 170 (69%) | 201 (75%) | 10 (32%) | P<0.01 |

*BMI: body mass index;; FiO_2_: inspired fraction of oxygen; ICU: intensive care unit; IQR: interquartile range; PaO_2_: arterial partial pressure of oxygen; SAPS II: Simplified Acute Physiology Score; Scr: serum creatinine; SD: standard deviation; SOFA:* *Sequential Organ Failure Assessment Score*

**Table S3: Comparison of AKI and non AKI patients**

| **Characteristics of patients** | **Non-AKI**  **(*n* = 200)** | **AKI**  **(*n* = 598)** | ***p* value** |
| --- | --- | --- | --- |
| Males, *n* (%) | 137 (68%) | 423 (71%) | 0.592 |
| Age (years), mean ± SD | 60 ± 13 | 63 ± 11 | **0.013** |
| BMI (kg/m²), mean ± SD | 29.7 ± 5.7 | 31 ± 6.9 | **0.022** |
| Chronic kidney disease, *n* (%) | 9 (4%) | 37 (6%) | 0.484 |
| Basal SCr (µmol/L), median [IQR] *(missing values, n = 470)* | 67 [55-80] | 76 [62-92] | **< 0.001** |
| Hypertension, *n* (%) | 88 (44%) | 314 (52%) | **0.041** |
| Diabetes, *n* (%) | 51 (25%) | 180 (30%) | 0.242 |
| Immunosuppression, *n* (%) | 13 (6%) | 24 (4%) | 0.255 |
| SAPS II | 35.21 ± 13.8 | 38.93± 15.3 | **<0.001** |
| Invasive mechanical ventilation* | 51 (26%) | 369 (62%) | **<0.001** |
| Intravenous fluid therapy* (per L) | 1.50 [1-2.3] | 1.38 ± [0.8-2.3] | 0.18 |
| SARS-CoV-2 Variant |  |  |  |
| *Wuhan, n (%)* | 114 (57%) | 402 (67%) | **< 0.001** |
| *Alpha, n (%)* | 49 (24%) | 105 (18%) |  |
| *Beta, n (%)* | 20 (10%) | 84 (14%) |  |
| *Gamma, n (%)* | 1 (0.5%) | 1 (0.2%) |  |
| *Delta, n (%)* | 16 (8%) | 6 (1%) |  |
| Dexamethasone, *n* (%) | 151 (75%) | 316 (53%) | **< 0.001** |
| Catecholamine use, *n* (%) | 37 (18%) | 335 (56%) | **< 0.001** |
| Length of intubation (days), median [IQR] | 7 [1.8-18.6] | 12.8 [7-20.8] | **< 0.01** |
| Prone position, *n* (%) | 65 (32%) | 405 (68%) | **< 0.001** |
| Length of sedation (days), median [IQR] | 3 [0.1-6.1] | 11 [5-19] | **< 0.01** |
| ICU length of stay (days), median [IQR] | 5 [2-9] | 11 [6-22] | **< 0.01** |
| ICU death, *n* (%) | 22 (11%) | 186 (31%) | **< 0.001** |

*BMI: body mass index; ICU: intensive care unit; IQR: interquartile range; SAPS II: Simplified Acute Physiology Score; SD: standard deviation; SOFA:* *Sequential Organ Failure Assessment Score *During the first 24 hours*

Among all descriptive variables in Table S3, only basal SCr variable had missing values. SCr missing values were estimated using the Modification of Diet in Renal Disease (MDRD) study equation, assuming that baseline eGFR is 75 mL/min/1.73m^2^.

**Table S4**

| Association between dexamethasone use and occurrence of AKI in patients admitted in an ICU for severe COVID-19 infection. Results of a cause-specific Cox model assuming that all patients admitted after 5^th^ July 2020 received DXM | | |
| --- | --- | --- |
|  | HR* | 95 CI |
| Dexamethasone use (vs no use) | 0.63 | 0.51 – 0.79 |
| *Adjusted for age (in years), sex (male/female), BMI (in kg/m²), CKD before ICU admission (yes vs no), hypertension before ICU admission (yes vs no), history of diabetes before ICU admission (yes vs no), immunodepression before ICU admission (yes vs no), invasive mechanical ventilation in the first 24 hours (yes vs no), Intravenous fluid therapy in the first 24 hours (per L), Catecholamine use in the first 24 hours (yes vs no), SAPS II (per unit), the different waves (first/second/third and fourth)  *BMI : Body Mass Index; CKD : Chronic kidney Disease; SAPS II : Simplified Acute Physiology Score* | | |

**Table S5**

Association between dexamethasone use and occurrence of AKI in patients admitted in an ICU for severe COVID-19 infection : sensitivity analysis, which included only patients with baseline serum creatinine data (not estimated baseline creatinine data), (n=328).

|  | HR* | 95 CI |
| --- | --- | --- |
| Dexamethasone use (vs no use) | 0.65 | 0.49 – 0.94 |

*Adjusted for age (in years), sex (male/female), BMI (in kg/m²), CKD before ICU admission (yes vs no), history of diabetes before ICU admission (yes vs no), immunodepression before ICU admission (yes vs no), invasive mechanical ventilation in the first 24 hours (yes vs no), Intravenous fluid therapy in the first 24 hours (per L), Catecholamine use in the first 24 hours (yes vs no), SAPS II (per unit), the different waves (first/second/third and fourth)

*BMI : Body Mass Index; CKD : Chronic kidney Disease; SAPS II : Simplified Acute Physiology Score*
